# CoIN: a network analysis for document triage

**DOI:** 10.1093/database/bat076

**Published:** 2013-11-11

**Authors:** Yi-Yu Hsu, Hung-Yu Kao

**Affiliations:** Department of Computer Science and Information Engineering, National Cheng Kung University, No. 1, University Road, Tainan City 701, Taiwan, R.O.C. (Republic of China)

## Abstract

In recent years, there was a rapid increase in the number of medical articles. The number of articles in PubMed has increased exponentially. Thus, the workload for biocurators has also increased exponentially. Under these circumstances, a system that can automatically determine in advance which article has a higher priority for curation can effectively reduce the workload of biocurators. Determining how to effectively find the articles required by biocurators has become an important task. In the triage task of BioCreative 2012, we proposed the Co-occurrence Interaction Nexus (CoIN) for learning and exploring relations in articles. We constructed a co-occurrence analysis system, which is applicable to PubMed articles and suitable for gene, chemical and disease queries. CoIN uses co-occurrence features and their network centralities to assess the influence of curatable articles from the Comparative Toxicogenomics Database. The experimental results show that our network-based approach combined with co-occurrence features can effectively classify curatable and non-curatable articles. CoIN also allows biocurators to survey the ranking lists for specific queries without reviewing meaningless information. At BioCreative 2012, CoIN achieved a 0.778 mean average precision in the triage task, thus finishing in second place out of all participants.

**Database URL:** http://ikmbio.csie.ncku.edu.tw/coin/home.php

## Introduction

With the increasing number of research studies in the medical community, the work of biocuration has become time-consuming with low efficiency. The curation of these abundant biomedical studies leads biocurators into a series of unanticipated problems: the related literature is usually large in number and unstructured in nature. To obtain high-quality information, databases usually require manual curation. Even with their best efforts, biocurators can only annotate a limited number of articles. Constraints, such as the limited number of biocurators, the rapid growth of biomedical literature and the compatibility of the resulting data formats, create barriers to the biocuration process (1).

When manual curation databases, such as PharmGKB (2) and the Comparative Toxicogenomics Database (CTD) (3), were developed, the biocurators used PubMed and Medical Subject Headings (MeSH) to understand and annotate medical terms. PubMed and MeSH are developed by the National Library of Medicine and contain a huge amount of literature, experimental results and ontology information. Determining how to effectively find the articles required by biocurators has therefore become an important task. For example, the Article Classification Task (ACT) in the BioCreative (Critical Assessment of Information Extraction Systems in Biology) competition aims to classify articles that are relevant to protein–protein interaction (PPI) curation (4). Thus, the ACT is helpful for building annotated PPI databases. However, there are many problems in the workflow of biocuration. The workflow of biocuration is a heuristic learning procedure, and the heuristic rules are based on the experience of the biocurators (5); that is, different biocurators might annotate differently. During the biocuration process, the biocurators annotate the chemicals based on their domain knowledge. To understand the behavior of biocurators, the BioCreative 2012 committee examined the decisions made by biocurators when they were determining whether articles should be curated. The decision is easily understood by biocurators, but the task is difficult for computers. Therefore, more studies must be conducted to ascertain the effects of text-mining approaches in classifying important articles.

Up to now, most researchers focused on the issue of relation extraction in the ACT, and only a few works considered the issue of multiple relation extraction. Therefore, we constructed an entity co-occurrence analysis system, which is applicable to PubMed articles and suitable for gene, chemical and disease queries. The relationship between two terms is often correlated to their co-occurrence in a sentence. The co-occurrence of two terms means that these two terms both occur in the same article. This co-occurrence determines the semantic relations between these two terms, although it does not guarantee that the two terms are indeed related. For example, ‘dementia’ and ‘atopic dermatitis’ might co-occur in the same article, but the reasons for the co-occurrence of the two terms are unclear. Co-occurrence features are still useful for providing possible candidates for relation extraction.

In this work, we propose a text-mining platform, known as the Co-occurrence Interaction Nexus (CoIN), to distill the entity co-occurrence information from literature and to measure the relationships between entities using a networking approach. CoIN integrated several named entity recognition tools and parsed single sentences in articles. The system is able to obtain co-occurrence pairs and create a ranking list. We assumed that network centralities represent the importance of co-occurrence pairs and would be useful in building an automatic curation system. Therefore, we calculated the mean average precision (MAP) from co-occurrence features and network centralities. The advantage of CoIN is that biocurators can survey the ranking lists of specific queries without reviewing meaningless information; thus, CoIN allows biocurators to focus on more useful tasks.

Network analysis concerns the relationships between processing entities. For example, the nodes in a social network are people, and the links are the friendships between the nodes. If we apply these concepts to the ACT, PubMed articles are the nodes, while the co-occurrences of gene–disease, gene–chemical and chemical–disease relationships are the links. Network analysis provides a visual map and a graph-based technique for determining co-occurrence relationships. These graphical properties, such as size, degree, centralities and similar features, are important. By examining the graphical properties, we can gain a global understanding of the likely behavior of the network. For this purpose, this work focuses on two themes concerning the applications of biocuration: using the co-occurrence–based approach to obtain a normalized co-occurrence score and using the network-based approach to measure network properties, e.g. betweenness and PageRank. CoIN integrates co-occurrence features and network centralities when curating articles. The proposed method combines the co-occurrence frequency with the network construction from text. The co-occurrence networks are further analyzed to obtain the linking and shortest path features of the network centralities.

We have organized the rest of this work as follows. The second section is a review of the literature and addresses related works in biocuration. The third section describes the system and methodology of CoIN. The fourth section is an evaluation and discussion. We conclude the article with conclusions and future work.

## Related work

Several studies investigated methods for extracting relations from biomedical literature. The intuitive methods use predefined phrase patterns or the co-occurrences of two queries from the text. However, these methods are limited by predefined knowledge patterns and are incapable of discovering new patterns. Therefore, machine learning (ML) techniques provide a better understanding when trying to discover new patterns. Hence, ML approaches have been widely used and have gained popularity in recent years (6, 7). To date, support vector machines (SVMs), k-nearest neighbor, Naive Bayes, decision trees and neural networks have been used to extract knowledge patterns (6, 8). Recently, attention has shifted from ML methods to natural language processing (NLP). NLP emphasizes linguistic features that are obtained from the text and can also distill knowledge patterns (9–12). Although the above strategies considered pattern extraction, a few works have been published on article classification.

The co-occurrence frequency can be considered a measurement that describes the overlapping characteristics between concepts. Therefore, the co-occurrence–based approach evaluates the curated relatedness of articles by exploring the co-occurrence frequency of different concept pairs. Curatable articles correlate with a high frequency of concept pairs, whereas non-curatable articles correlate with a low frequency of concept pairs. The shortest paths on the graphs are examined for various forms (13). Problems arise when many concepts share the same information content, which leads to redundant concept pairs. Several studies applied concepts with different information content to approximate the curated relatedness (14, 15). Wilbur and Yang retrieved articles from PubMed and transformed them into a matrix; the matrix was used to correlate the documents with their term frequency (16). Moreover, the researchers evaluated the curated relatedness of terms using the co-occurrences of terms. Patwardan and Pedersen used a context vector to estimate the value of curated relatedness (17) and constructed the context vector from the literature by word sense discrimination (18) and latent semantic indexing (19). However, the co-occurrences of two concepts do not correspond to the actual curated relatedness. Bollegala *et al.* proposed a framework for the curated relatedness between concepts; this framework combines the lexical patterns from short text snippets with four characteristics of page counts. Then, the authors used clustering lexical patterns to improve the performance (20).

Recent computational biology research has suggested that network-based approaches may indeed facilitate processes that are beneficial to the understanding of molecular biology. These benefits include integration of heterogeneous databases, prediction of disease genes and increased quality of modules of cellular machinery. Eronen and Toivonen developed a system with protein interaction prediction and a disease gene prioritization task as instances of link predictions, and the predictions were based on a proximity measure of the integrated graph (21). Then, Winter *et al.* reported that identifying prognostic genes connects gene expression measurements to a network of known relationships. The researchers ranked the genes using both expression and network information in a manner similar to Google’s PageRank (22). At the same time, Atias and Sharan described a comparative analysis of networks from multiple species that detected significant biological patterns and provided more interpretation (23).

Several studies use network centralities to make tentative predictions of important vertices in compound networks. In this case, network centralities are able to measure the global influence of individual proteins. Jeong *et al.* reported that the proteins with a high degree in PPI networks may be important proteins (24). Yu *et al.* demonstrated that proteins with high betweenness centrality are important proteins in yeast PPI networks (25). More recently, the increase in network approaches for studying heterogeneous networks has decreased the accuracy due to incomplete networks and noise.

In recent years, interest in the issue of assisting manual curation has dramatically increased (26). Manual curation plays an important role in supporting basic analyses for advanced research, and BioCreative 2012 focused on the integration of biocuration. The BioCreative 2012 subcommittee identified three areas, or tracks, that comprised independent but complementary aspects of data curation (27). The three areas are literature triage (Track I); curation workflow (Track II); and text mining/NLP systems (Track III). Track I participants developed systems that would effectively triage and prioritize articles for curation. CoIN produced notable results in BioCreative 2012 Track I.

## System description

CoIN is a web-based system that assists biocurators in assessing articles according to the correlations of their terms among sentences. For searching relevant documents, CoIN adopts the co-occurrence features and co-occurrence networks from PubMed articles, and then CoIN applies network analyses to distinguish between curatable and non-curatable articles. Although co-occurrence features are the basic components of searching for the patterns of domain knowledge, there are still many restrictions on named entity recognition; training set problems, such as imbalance and shortage, are of particular concern.

Network analysis has been applied to different research topics, such as phylogenetics, function predictions, human diseases and drug development (28, 29). At the same time, the pre-tagging results of CoIN were developed based on the state-of-the-art named entity recognition tools in BioCreative III (30, 31). Furthermore, we collected dictionary corpora to recognize disease and chemical names during sentence-level processing. Therefore, the idea of CoIN is basically generated from the co-occurrence of gene, disease and chemical names in a specific article.

## Curation workflow

**Figure 1.**
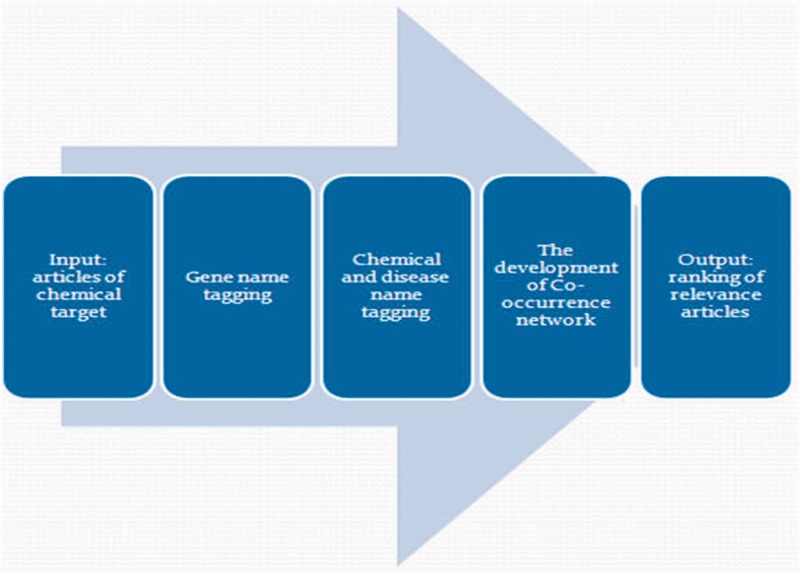
The workflow of CoIN.

**Figure 2.**
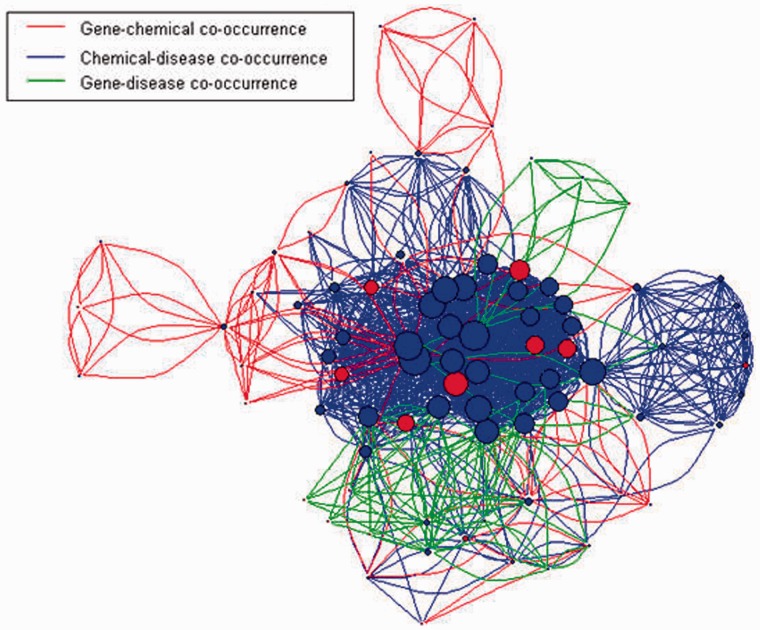
Co-occurrence interaction network of urethane from the test set. Vertices are PubMed articles, and the size of each vertex is specified by the degree of the vertex. Blue vertices are curatable articles, whereas red vertices are non-curatable articles. Red, blue and green edges are established when two PubMed articles have the co-occurrences of gene–chemical, chemical–disease and gene–disease relationships, respectively.

For the convenience of biocurators, CoIN allows users to query genes, diseases and chemicals. As shown in Figure 1, CoIN uses AIIAGMT (32) to identify gene names and separate articles into sentences. Next, we train conditional random fields to predict chemical names in the articles, and the training patterns are extracted from the CTD. This statistical modeling method is frequently applied in pattern recognition. To tag disease names, CoIN uses a dictionary-based method to identify diseases, and the dictionary is also extracted from the CTD. After collecting the tagging names, CoIN calculates the co-occurrences of the tagging names for each sentence. Then, the co-occurrence network is constructed using the co-occurrences of gene–disease, gene–chemical and chemical–disease relationships, as shown in Figure 2. In the last stage of CoIN, the system provides the normalized co-occurrence score, the betweenness and the PageRank value for prioritizing PubMed articles. In the ‘Methods’ section, we introduce the normalized co-occurrence score, betweenness and PageRank.

**Figure 3.**
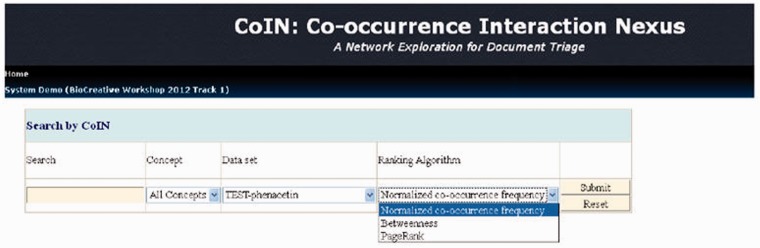
The input screen of CoIN.

**Figure 4.**
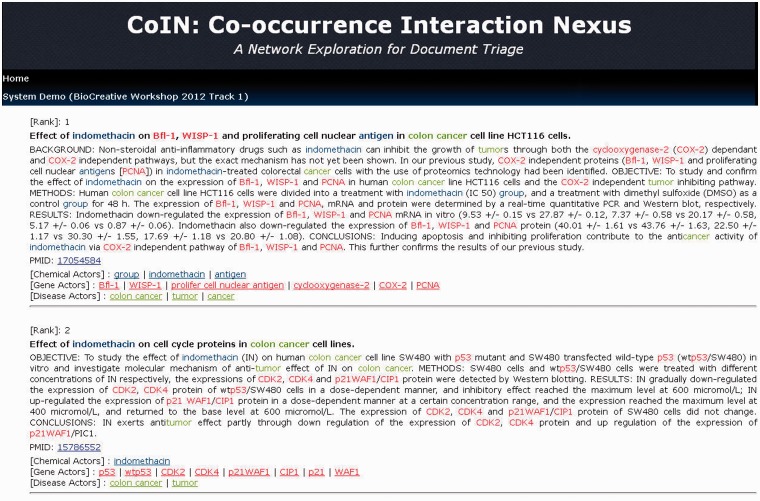
The output screen of CoIN.

For example, we use the PubMed articles for phenacetin as an input list; otherwise, the user can input a gene, disease or chemical name, as shown in Figure 3. After the computation is finished, we can obtain a ranking list, as shown in Figure 4. The name recognition process is usually time-consuming for the system schema of CoIN. CoIN provides a quick sorting result to biocurators after the name recognition process is finished. CoIN takes less time to train complex features, but the system immediately returns the ranking result from the network centralities of co-occurrence networks.

Many interaction data are accompanied by significant noise, and an overestimation is caused by the overlapping interactions. The noise stems from the related problem of named entity recognition. For example, we processed gene, chemical and disease names as single entities. However, various gene, chemical and disease names consisting of multiple words are not separable during parsing. Consequently, the named entity recognition is restricted by the entity anonymization. Furthermore, there are many chemicals describing the curative effect in the same sentence, but these chemicals are usually synonyms. In this case, the combination relationships of these chemicals also become noise.

## Methods

### Normalized co-occurrence score

In analyzing the training data set, we found that the disease entity recognition rate was low. Therefore, we designed the normalized co-occurrence score to adjust the influence of the imbalanced recognition rate. Using a normalized co-occurrence score avoids many frequent patterns for describing overlapped combinations. Removing such patterns may result in a performance decrease, and these patterns might be insignificant for the ACT. However, we found that the number of true positives for the normalized co-occurrence score increased when the disease names were not treated as a single entity, but this finding was not applied to the official runs. The frequencies of co-occurrence of gene–disease, gene–chemical and chemical–disease are normalized by the standard score z as follows.
(1)
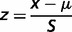

where

*x* is the co-occurrence frequency of either the gene–disease, gene–chemical or chemical–disease relationship;

*μ* is the mean value of a set of *x*; and

*S* is the standard deviation of a set of *x.*

After calculating the standard score z for the gene–disease, gene–chemical and chemical–disease frequency in an article, we define the sum of the above three standard scores of z as the normalized co-occurrence score of the article.

### Betweenness

CoIN applies betweenness centrality to measure the importance of vertices (articles) in a network. The betweenness centrality is equal to the number of shortest paths from all vertices to all others that pass through that vertex. A high betweenness centrality means that vertices have a high probability of occurring on a randomly chosen shortest path between two randomly chosen vertices (33). The betweenness centrality of a vertex *v* in a network is calculated as follows (34).
(2)
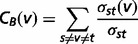

where


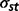
 is the total number of shortest paths from node *s* to node *t*; and


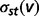
 is the number of those paths that pass through the vertex.

### PageRank

PageRank is a famous linking algorithm, which was developed by the founder of Google (35). To rank each web page, the in-links (inward-directed edges) and out-links (outward-directed edges) are calculated. An in-link is a hyperlink that other web pages use to direct people to the linked web page, and an out-link allows people to access other web pages. The PageRank algorithm ranks the page by the linking structure of networks via a random walking model. For any vertex *V* in a network, the PageRank value is calculated as follows.
(3)
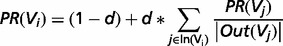

where

d is a damping factor and is set to 0.85;

*PR*(*V_i_*) is the PageRank value of *V_i_*;

In(*V_i_*) are the in-links of *V_i_*; and

Out(*V_j_*) are the out-links of *V_j_*.

**Figure 5.**
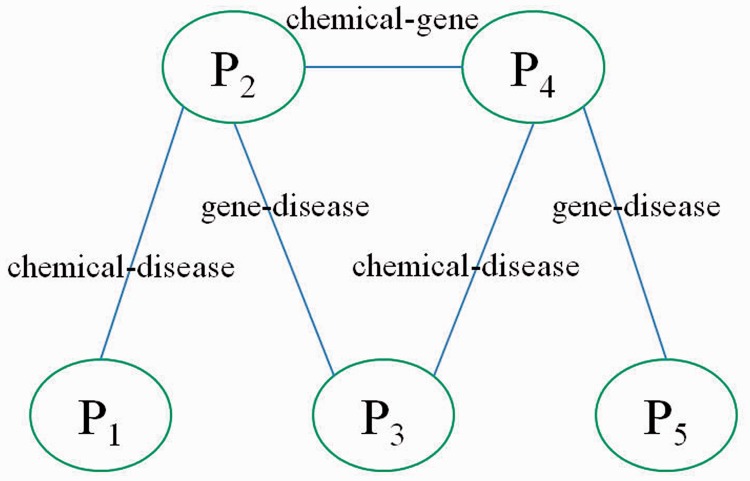
A toy example of a co-occurrence interaction network. P_i_ is a PubMed article. If any sentence shares a co-occurrence of a chemical–disease, chemical–gene and gene–disease relationship between two articles, an edge is established.

After computing the *PR* value of the vertices in networks, we can consider that the vertices with a higher *PR* value have more influence than the vertices with a lower *PR* value. The co-occurrence network is constructed using the information of gene–disease, gene–chemical and chemical–disease co-occurrences, as shown in Figure 5. The co-occurrence network is derived from the linking structure of web pages, but the co-occurrence relationships are essentially different from the in- and out-links. In Figure 5, we use an undirected edge to represent the co-occurrence relationship between entities and this edge also represents a bidirectional edge when counting in- and out-degree of a node in the undirected network. Thus, the co-occurrence network is displayed as an undirected graph, where vertices represent PubMed articles and edges represent co-occurrence interactions between PubMed articles. Note that both the in- and out-links of vertex i and vertex j are increased by 1 if there is an edge between vertex i and vertex j. For example, P_2_ and P_4_ have the highest betweenness value [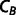
(P_2_) = 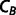
(P_4_) = 3], while 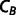
(P_1_) = 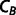
(P_3_) = 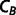
(P_5_) = 0. At the same time, P_2_ and P_4_ also have the highest PageRank value [*PR*(P_2_) = *PR*(P_4_) = 0.29], while *PR*(P_3_) = 0.19 and *PR*(P_1_) = *PR*(P_5_) = 0.11. The results of this toy example show that P_2_ and P_4_ are more important than the other nodes in the co-occurrence network.

When the co-occurrence features and network centralities of PubMed articles are evaluated, we define CoIN_index_ to estimate the relevance scores of PubMed articles. A concept pair means that two named entities occur within a single sentence in a document. Note that a neighbor of a concept pair (C_i_) is also another concept pair that co-occurs with C_i_ in the same document. CoIN_index_ calculates the concept pairs in data sets, and the neighbors of concept pairs are collected. After retrieving the neighbors of concept pairs, we construct co-occurrence networks, and these networks are the linking structures of concept pairs. Furthermore, we use linear combination to adjust the co-occurrence model and the network-based model. The co-occurrence model outputs the two scores of co-occurrence for each article. In contrast to the co-occurrence model, the network-based model includes the PageRank and the betweenness in the same manner. After calculating the scores from the co-occurrence model and the network-based model, we combine these two scores into the CoIN_index_ for classifying curated articles. However, we consider a constant α, which is a damping factor, to determine the weight of concept pairs. We compute their CoIN_index_ using the following equation:
(4)
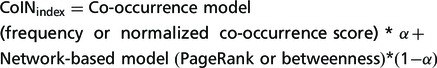



### Data set

The training data set of the triage task for the BioCreative 2012 was distributed to participants (http://www.biocreative.org/tasks/bc-workshop-2012/Triage/). The data set was critical for participants to understand the CTD curation process; thus, the data set that consisted of 1725 articles had been previously triaged and curated by the CTD biocurators. The training data set was organized in a series of input files that contained all associated curated data for eight target chemicals (raloxifene, aniline, amsacrine, doxorubicin, aspartame, quercetin, 2-acetylaminofluorene and indomethacin). The test data set that consisted of 444 articles was collected from three target chemicals: urethane, phenacetin and cyclophosphamide (27).

## Evaluation

To assess the performance and for comparison with other methods, we use the MAP to calculate the performance score. MAP represents the mean of the average precisions (denoted as AP) over the set of queries. In this work, the MAP score calculations indicate that an article was counted as curated if it had one or more associated curated interactions. We briefly introduce the evaluation metrics as follows. In the field of information retrieval, precision is the part of retrieved documents that are relevant to the query:
(5)
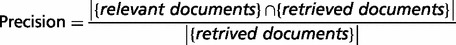

The AP evaluates the ranking of relevant documents that are retrieved and compares the search performance. AP can be defined using the following equation:
(6)
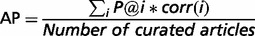

where *i* is the rank, *corr*(□) is a binary function for the relevance of a given rank and *P@i* is the precision at a given cutoff rank. MAP can be defined as follows:
(7)
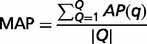

where *Q* is the number of queries.

**Figure 6.**
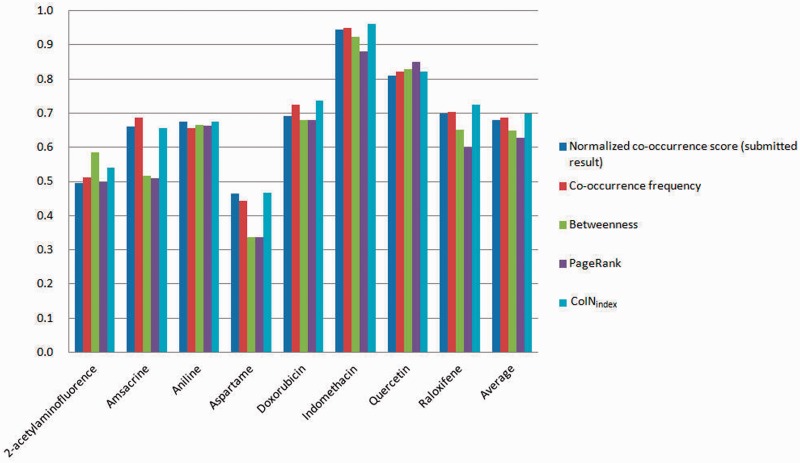
MAP of the training set.

We submitted the normalized co-occurrence score for the triage task, but we also assessed the training set and test set using other approaches. For the training set, both the co-occurrence–based approach and the network-based approach may have contributed to the fact that approximately half of the target chemicals were difficult to curate (<70%), in particular aspartame, as shown in Figure 6—that is, the co-occurrence relations may not be the key points in which biocurators are interested. Figure 6 shows that the vertices with a high betweenness and PageRank value obviously occupy the important positions in a network. Therefore, the vertices with a high betweenness and PageRank value are more likely to be important articles, and the ranking result indicates that the network-based approaches outperform the co-occurrence–based approaches. However, when we consider the tagging behavior of biocurators, the co-occurrence-based approaches provide more opportunities to curate terms intuitively – that is, the co-occurrence-based approaches find more unknown patterns than those found by the network-based approaches. On the other hand, the overestimated combinations in a sentence also easily produce noise. To avoid bias, network-based approaches are suitable for evaluating more complex interaction networks.

**Figure 7.**
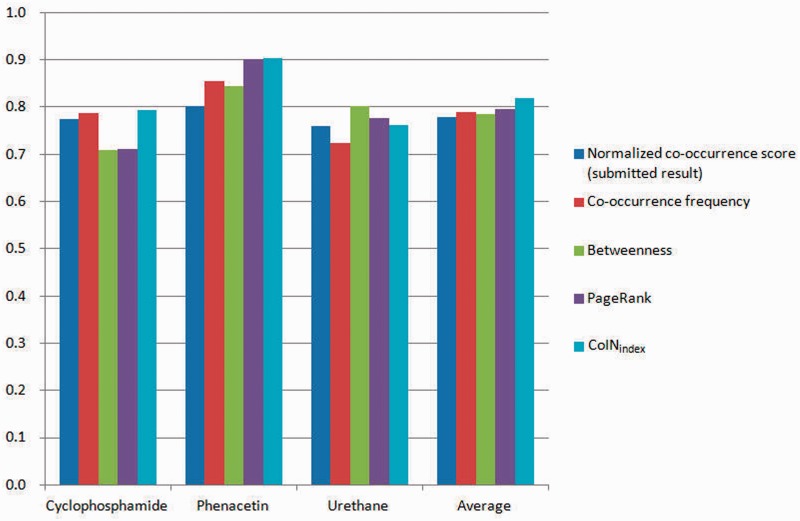
MAP of the test set.

For the test set, we did not analyze and optimize for the submission. According to our training set experiments, classification performance is sensitive to the combinations of co-occurrence pairs. For the submitted run, we applied the normalized co-occurrence score because we found that the rate of recognizing disease names was underestimated—that is, we assumed that the normalized co-occurrence score provided robustness against the low rate of entity recognition. The MAP of the submitted run is 0.778. We received second place in the triage task of BioCreative 2012, and the best MAP score was 0.803 (36). The scores of test target chemicals and approaches are illustrated in Figure 7. The ranking lists of network-based approaches are better than those of the co-occurrence–based methods, and the MAP of PageRank is 0.796, which is the best result from four approaches. Furthermore, we applied CoIN_index_ to curate the test and training sets. When the co-occurrence model and the network-based model used the frequency and PageRank, respectively, the best performance was 0.819 for the test set and 0.698 for the training set, respectively (α = 0.1, α = 0.3). Note that the network-based approaches apply the linking structure between the frequency of concept pairs and the co-occurrence networks. However, the linking structure of co-occurrence does not affect the performance. Hence, the average MAP of PageRank is slightly superior to that of the co-occurrence frequency.

## Discussion

In the discussion, we further investigated the relations between co-occurrence features and biocuration. The work was not fully optimized at the time of the competition due to time constraints. However, after analyzing a series of BioCreative data sets, we believe further improvement is possible based on ML techniques and also on the recently released gold standard test set. However, ML techniques led to an overfitting problem and decreased the classification performance in the test set, particularly for the triage task. Therefore, our tuning strategy for CoIN_index_ was focused on different data and feature combinations, not on the tuning parameters and heuristic rules. Our system shows that the strategy of using both co-occurrence and network features in our classification framework is a good combination for the triage task.

**Figure 8.**
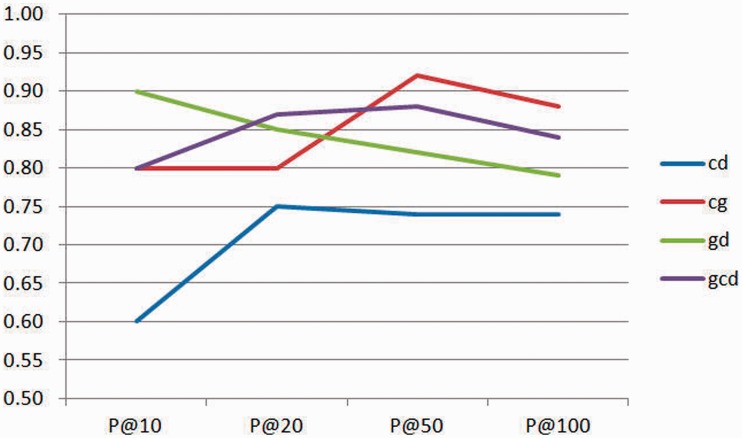
P@k of training set. cd: chemical–disease relations co-occur in a sentence, cg: chemical–gene relations co-occur in a sentence, gd: gene–disease relations co-occur in a sentence, gcd: the total occurrence of gd, cd and cg relations.

To understand the importance of co-occurrence features in the triage task, we used the training set to compute P@10, P@20, P@50 and P@100, as shown in Figure 8. The precision can be evaluated at a given cutoff of ranking, and we can consider only the top k results returned by the system, known as P@k. We used the number of chemical–disease (cd), chemical–gene (cg), gene–disease (gd) relations and the sum of three co-occurrence features (gcd) to measure the precision of CoIN at different values of k. In Figure 8, we can see that the cd and gd pairs decrease the precision of gcd, especially at P@20 and P@100. The exact reason for the low recognition rate of disease names is unclear, but the major problem in identifying disease names is that researchers tend to use general English terms, not MeSH terms. We found that the overall frequency of different concept pairs was effective for our ranking-based search system. However, our current performance is also limited by the recognition capability. It is recommended that co-occurrence features be regarded as an important feature in biocuration. In addition, there are problems in entity recognition for chemicals and diseases, and this problem has much room for improvement.

The observed differences between curated information led us to further research the influence of specific factors of the co-occurrence structure of curated articles. An independent sample t test was conducted to evaluate the hypothesis that there are more co-occurrence pairs in curatable articles than in non-curatable articles. The hypothesis was significant (*P* < 0.05). As shown in Table 1, for the different characteristics that were retrieved using our approaches, on average, there were more co-occurrence pairs in curatable articles than in non-curatable articles. It is reasonable to believe that there is a significant difference between the two groups in their performance—that is, the statistical evidence suggests that there are some co-occurrence relations between curatable and non-curatable articles.

**Table 1.** Characteristics of the curated articles

Features	*P*-value of curated and non-curated articles
Gene–disease	2.20*10^−16^
Chemical–disease	1.17*10^−11^
Chemical–gene	9.71*10^−10^
Gene–disease–chemical	2.20*10^−16^
Z-standard-total	2.20*10^−16^
Betweenness	9.32*10^−6^
PageRank	2.20*10^−16^

As discussed above, the statistics of co-occurrence raised the possibility that biocurators might annotate the articles in the same manner. In our system, the development of the network-based model is closely tied to the effective use of the co-occurrence model. Hence, we used co-occurrence and network features to train SVM models, and then the SVM classifiers applied different features to the training and test sets. Note that we used an SVM with a linear kernel and trained SVM models in a 5-fold cross-validation. Table 2 presents the effect of applying an SVM using co-occurrence and network features on the BioCreative 2012 Triage task. After training the classifiers with the training set, we used the training and test sets to make predictions. As shown in Table 2, adding network features to the co-occurrence features boosts the performance in the test set, and the overall co-occurrence frequency also enhances the performance. For SVM classifiers, there is less improvement; however, it demonstrates that network features provide a positive effect for the ACT. For the BioCreative 2012 Triage task, possible feature candidates, including both co-occurrence and network features, were examined and explored. As a result, the overall co-occurrence frequency and PageRank were further selected for better triage. In addition, betweenness is helpful to increase the precision and recall in the test set but decreases the recall in the training set.

**Table 2.** Overall performance for co-occurrence and network feature combinations

Features	Training data set			Test data set		
	Precision	Recall	F-measure	Precision	Recall	F-measure
cd + cg + gd	0.716	0.792	0.752	0.665	0.856	0.748
cd + cg + gd + betweenness	0.720	0.783	0.750	0.671	0.867	0.757
cd + cg + gd + PageRank	0.711	0.775	0.742	0.714	0.845	0.774
cd + cg + gd + gcd	0.713	0.811	0.759	0.664	0.853	0.746
cd + cg + gd + gcd + betweenness	0.719	0.788	0.752	0.669	0.863	0.754
cd + cg + gd + gcd + PageRank	0.710	0.811	0.757	0.714	0.863	0.782

Co-occurrence features: ‘cd’ means the number of chemical–disease relations, ‘cg’ means the number of chemical–gene relations, ‘gd’ means the number of gene–disease relations and ‘gcd’ means the total occurrence of gd, cd and cg. The network features alternate from betweenness to PageRank.

## Conclusion and future work

In this study, we used co-occurrence– and network-based approaches to develop a system (CoIN) and evaluated the ranking of the CTD data sets. CoIN applies the co-occurrences of sentence structures and the linking activities between biomedical terms, such as genes, chemicals and diseases, to prioritize the importance of articles. Note that our approach is different from traditional supervised learning methods. CoIN begins with the automatic identification of named entity recognition and connections in the neighbors. Then, we constructed heterogeneous co-occurrence networks from the combinations of different concept pairs. Next, we computed the co-occurrence frequency and network centralities for concept pairs. If an article has more concept pairs, this article has a higher priority to be curated. Finally, we tested CoIN with the test data, and CoIN demonstrated its ability to curate articles. The experiments with the test data showed that the network-based approaches perform better than the co-occurrence–based approaches. The proposed system is also helpful for biocurators to customize their own curation patterns.

In the future, we hope to clarify the influence of concept pairs in CoIN. Although we have investigated gene–disease, gene–chemical and chemical–disease concept pairs, we believe that more detailed research focusing on the relations of concept pairs will improve the performance of CoIN.

## References

[1] Kim S, Wilbur W (2011). Classifying protein-protein interaction articles using word and syntactic features. BMC Bioinformatics.

[2] Hernandez-Boussard T, Whirl-Carrillo M, Hebert JM (2008). The pharmacogenetics and pharmacogenomics knowledge base: accentuating the knowledge. Nucleic Acids Res..

[3] Mattingly CJ, Colby GT, Forrest JN (2003). The comparative toxicogenomics database (CTD). Environ. Health Perspect.

[4] Arighi CN, Roberts PM, Agarwal S (2011). BioCreative III interactive task: an overview. BMC Bioinformatics.

[5] Pedersen T, Pakhomov SVS, Patwardhan S (2007). Measures of semantic similarity and relatedness in the biomedical domain. J. Biomed. Inform..

[6] Donaldson I, Martin J, de Bruijn B (2003). PreBIND and Textomy - mining the biomedical literature for protein-protein interactions using a support vector machine. BMC Bioinformatics.

[7] Mitsumori T, Murata M, Fukuda Y (2006). Extracting protein-protein interaction information from biomedical text with SVM. IEICE—Trans. Inf. Syst..

[8] Sætre R, Yoshida K, Miwa M (2010). Extracting protein interactions from text with the unified AkaneRE event extraction system. IEEE/ACM Trans. Comput. Biol. Bioinformatics.

[9] Tsuruoka Y, Miwa M, Hamamoto K (2011). Discovering and visualizing indirect associations between biomedical concepts. Bioinformatics.

[10] Stenetorp P, Topić G, Pyysalo S (2011). BioNLP Shared Task 2011: supporting resources. Proceedings of the BioNLP Shared Task 2011 Workshop.

[11] Faro A, Giordano D, Spampinato C (2012). Combining literature text mining with microarray data: advances for system biology modeling. Brief. Bioinformatics.

[12] Schneider G, Clematide S, Rinaldi F (2011). Detection of interaction articles and experimental methods in biomedical literature. BMC Bioinformatics.

[13] Caviedes JE, Cimino JJ (2004). Towards the development of a conceptual distance metric for the UMLS. J. Biomed Inform..

[14] Lin D (1998). Automatic retrieval and clustering of similar words. Proceedings of the 17th international conference on Computational linguistics - Volume 2.

[15] Jiang JJ, Conrath DW (1997). Semantic similarity based on corpus statistics and lexical taxonomy. International Conference Research on Computational Linguistics (ROCLING X).

[16] Wilbur WJ, Yang Y (1996). An analysis of statistical term strength and its use in the indexing and retrieval of molecular biology texts. Comput. Biol. Med..

[17] Patwardhan S, Pedersen T (2006). Using {WordNet}-based context vectors to estimate the semantic relatedness of concepts. Proceedings of the EACL 2006 Workshop Making Sense of Sense—Bringing Computational Linguistics and Psycholinguistics Together.

[18] Schütze H (1998). Automatic word sense discrimination. Comput. Linguist..

[19] Deerwester S, Dumais S, Furnas G (1990). Indexing by latent semantic analysis. J. Am. Soc. Inform. Sci..

[20] Bollegala D, Matsuo Y, Ishizuka M (2011). A web search engine-based approach to measure semantic similarity between words. IEEE Trans. Knowl. Data Eng..

[21] Eronen L, Toivonen H (2012). Biomine: predicting links between biological entities using network models of heterogeneous databases. BMC Bioinformatics.

[22] Winter C, Kristiansen G, Kersting S (2012). Google goes cancer: improving outcome prediction for cancer patients by network-based ranking of marker genes. PLoS Comput. Biol..

[23] Atias N, Sharan R (2012). Comparative analysis of protein networks: hard problems, practical solutions. Commun. ACM.

[24] Jeong H, Mason SP, Barabasi AL (2001). Lethality and centrality in protein networks. Nature.

[25] Yu H, Kim PM, Sprecher E (2007). The importance of bottlenecks in protein networks: correlation with gene essentiality and expression dynamics. PLoS Comput. Biol..

[26] Neveol A, Islamaj Dogan R, Lu Z (2011). Semi-automatic semantic annotation of PubMed queries: a study on quality, efficiency, satisfaction. J. Biomed. Inform..

[27] Wiegers TC, Davis AP, Mattingly CJ (2012). Collaborative biocuration—text-mining development task for document prioritization for curation. Database.

[28] Gavin A-C, Bosche M, Krause R (2002). Functional organization of the yeast proteome by systematic analysis of protein complexes. Nature.

[29] Furney S, Alba MM, Lopez-Bigas N (2006). Differences in the evolutionary history of disease genes affected by dominant or recessive mutations. BMC Genomics.

[30] Huang M, Liu J, Zhu X (2011). GeneTUKit: a software for document-level gene normalization. Bioinformatics.

[31] Wei CH, Kao HY (2011). Cross-species gene normalization by species inference. BMC Bioinformatics.

[32] Hsu C-N, Chang Y-M, Kuo C-J (2008). Integrating high dimensional bi-directional parsing models for gene mention tagging. Bioinformatics.

[33] Freeman L (1977). A set of measures of centrality based upon betweenness. Sociometry.

[34] Brandes U (2001). A faster algorithm for betweenness centrality. J. Math. Sociol..

[35] Brin S, Page L (1998). The anatomy of a large-scale hypertextual web search engine. Seventh International World-Wide Web Conference (WWW 1998).

[36] Kim S, Kim W, Wei C-H (2012). Prioritizing PubMed articles for the comparative toxicogenomic database utilizing semantic information. Database.

